# Changes in vaginal microbiome after focused ultrasound treatment of high-risk human papillomavirus infection-related low-grade cervical lesions

**DOI:** 10.1186/s12879-022-07937-8

**Published:** 2023-01-05

**Authors:** Wenping Wang, Yujuan Liu, Yamei Yang, Jiaojiao Ren, Honggui Zhou

**Affiliations:** Department of Gynecology, Affiliated Hospital of North Sichuan Medical College, Wenhua Road 63, Nanchong, 637000 China

**Keywords:** Focused ultrasound, High-risk human papillomavirus, Vaginal microbiome, Low-grade squamous intraepithelial lesion

## Abstract

**Background:**

In this study, the changes of vaginal microbiome after focused ultrasound (FU) treatment were evaluated to explore the possible mechanism of FU in the treatment of high-risk human papillomavirus (HR-HPV) infection.

**Methods:**

This study was nested in the FU arm of a prospective cohort study. A total of 37 patients diagnosed with HR-HPV infection-related cervical low-grade squamous intraepithelial lesion (LSIL) who met the inclusion criteria were enrolled in this study from October 2020 to November 2021, and these patients were treated with FU. We used 16S ribosomal RNA (16S rRNA) gene amplicon sequencing to profile the vaginal microbiota composition of patients before and 3 months after FU treatment.

**Results:**

After FU treatment, HR-HPV was cleared in 24 patients, with a clearance rate of 75.0% (24/32). *Lactobacillus iners* was the predominant species among all samples. No significant difference was found in alpha-diversity index before and 3 months after FU treatment (*P* > 0.05), but the rarefaction curves showed that the vaginal microbial diversity before FU treatment was higher than that after FU treatment. Linear discriminant analysis (LDA) effect size (LEfSe) showed that *Bifidobacterium* contributed the most to the difference between the two groups at the genus level, and the abundance after FU treatment was significantly higher than that before treatment (*P* = 0.000).

**Conclusions:**

The decrease of vaginal microbial diversity may be related to the clearance of HR-HPV infection, and FU treatment contributed to the decrease of vaginal microbial diversity. Increased *Bifidobacterium* abundance in the vaginal microbiome may be associated with clearance of HR-HPV infection, and FU treatment may contribute to the increase in *Bifidobacterium* abundance.

*Trial registration number:* This study was registered in the Chinese Clinical Trial Registry on 23/11/2020 (ChiCTR2000040162).

## Background

Human papillomavirus (HPV) remains one of the most important oncogenic DNA viruses and has been identified as the cause of approximately 5% of cancers worldwide [1]. To date, more than 200 HPV genotypes have been identified, of which about 15 high-risk genotypes are considered to be necessary causes of cervical cancer (CC) [2]. In addition, socio-environmental factors, epidemiological factors, and genetic factors related to the host are considered to be co-factors that act together with HPV to influence the risk of CC development [3–5]. Over the past decade, emerging evidence suggests that the vaginal microbiome also plays a role in HPV infection, persistence, and cervical carcinogenesis [6–8].

In the lower female reproductive tract (vagina and cervix), the predominance of *Lactobacillus* is associated with vaginal health, and the depletion of these microorganisms can lead to a number of adverse conditions, such as increased risk of acquiring sexually transmitted diseases (STDs), spontaneous miscarriage, preterm birth or pelvic inflammatory disease [9]. Bacterial vaginosis (BV), characterized by depletion of *Lactobacillus* and overgrowth of anaerobic bacteria, has been reported to be significantly associated with increased risk of HPV infection and decreased HPV clearance [10]. Meanwhile, several studies have shown that women with cervical intraepithelial neoplasia (CIN) and CC have a vaginal microbial profile of community state type (CST) IV, characterized by depletion of Lactobacillus spp. and a substantial increase in vaginal microbiome diversity compared to healthy women [8, 11, 12].

As a novel and noninvasive treatment technology, focused ultrasound (FU) has achieved good curative effect in the treatment of cervical lesions, and has attracted much attention in China. In economically underdeveloped regions like southwestern China, some women diagnosed with high-risk human papillomavirus (HR-HPV) infection-related cervical low-grade squamous intraepithelial lesion (LSIL) lack previous screening data, have limited access to follow-up, and therefore urgently require treatment rather than observation. Our previous studies have confirmed that FU can effectively eliminate HR-HPV infection and reverse LSIL lesions [13, 14]. However, the response of vaginal microbiota to FU treatment remains unknown. Therefore, the purpose of this study was to explore the changes in the vaginal microbiome following FU treatment, and to provide clues for the mechanism of FU in clearing HR-HPV infection from the perspective of vaginal microbiota.

## Methods

### Study population and sample collection

From October 2020 to November 2021, women who visited the gynecology clinic of the Affiliated Hospital of North Sichuan Medical College, Nanchong city, Southwest China, were enrolled in this study if they met the following inclusion criteria: (1) women aged 18–55 years with a sexual history, confirmed HR-HPV infection by HPV-DNA test, and cervical biopsy revealed histological LSIL; (2) non-pregnant, non-lactating and non-menstruating women; (3) no antiviral treatment and corticosteroids and sex hormones application within 3 months before sampling; (4) no systemic or vaginal topical antibiotic therapy or antifungal therapy within 1 month before sampling; (5) no vaginal douching and administration within 1 week before sampling; and (6) no sexual activity within 72 h before sampling. Women with acute inflammation of the reproductive tract, sexually transmitted infections (STIs), immunodeficiency diseases, severe cardiac, hepatic and renal dysfunction, coagulopathy, and those who cannot be followed up were excluded.

HPV testing was performed using a High-Risk Human Papillomavirus Genotyping Real Time PCR Kit (Shanghai ZJ Bio-Tech Co., Ltd. China), which tests for 15 HR-HPV genotypes (16, 18,31, 33, 35, 39, 45, 51, 52, 56, 58, 59, 66, 68, and 82). Cytological results were reported according to the 2014 Bethesda System [15], and histological diagnosis was obtained from two experienced pathologists using a 2-Tier Terminology as recommended by the LAST guidelines [16].

Samples for vaginal microbial analysis were collected from the upper third of the vaginal lateral wall using a sterile swab under direct visualization prior to FU treatment. The vaginal swab samples were immediately frozen and stored at − 80 °C until extraction. The sampling process at the 3-month follow-up after FU treatment was the same as above. The samples before and after FU treatment were collected during the follicular phase of the patients.

### Focused ultrasound treatment

Women were treated with the Ultrasound Therapeutic Device (Model-CZF, Chongqing Haifu Medical Technology Co., Ltd. China). Women were placed in the lithotomy position and a sterile speculum was used to expose the cervix. The treatment probe was placed in close contact with the cervix, and a circular scanning was performed at the speed of 5–10 mm/s with the external cervical os as the center. The treatment typically lasted for 3–5 min until the lesion and the squamocolumnar junction shrunk with moderate inward depression of the external cervical os.

### DNA extraction and MiSeq sequencing

Microbial DNA was extracted using the FastDNA^®^ Spin Kit for Soil (MP Biomedicals, USA) according to the manufacturer’s instructions. All DNA samples were quality checked and the concentration was quantified by NanoDrop 2000 (Thermo Fisher Scientific, USA). Bacterial 16S rRNA gene fragments (V3–V4) were amplified from the extracted DNA using primers 338F (5′-ACTCCTACGGGAGGCAGCAG-3′) and 806R (5′-GGACTACHVGGGTWTCTAAT-3′). Amplification was performed in 20-μL reactions with TransStart Fast Pfu DNA Polymerase (TransGen Biotech, China), 5 μM of each primer and 10 ng of template. The reactions were performed under the following thermal profile: 95 °C for 3 min, followed by 25 cycles of 95 °C for 30 s, 55 °C for 30 s, and 72 °C for 45 s, and one cycle of 72 °C for 10 min and a 4 °C hold. PCR products were examined by 2% agarose gel electrophoresis and then purified using an AxyPrep DNA Gel Extraction Kit (Axygen Biosciences, USA). Amplicons were quantified using the Quantus™ Fluorometer (Promega, USA). Amplicons were subjected to paired-end sequencing on the Illumina MiSeq sequencing platform at Majorbio Bio-Pharm Technology Company (Shanghai, China).

### Sequence analysis

After demultiplexing, the resulting sequences were merged with FLASH (v1.2.11) [17] and quality filtered with fastp (0.19.6) [18]. Then the high-quality sequences were de-noised using DADA2 [19] plugin in the QIIME 2 [20] (version 2020.2) pipeline with recommended parameters, which obtained single nucleotide resolution based on error profiles within samples. DADA2 denoised sequences were usually called amplicon sequence variants (ASVs). Taxonomic assignment of ASVs was performed using the Naive baye consensus taxonomy classifier implemented in QIIME 2 and the Silva 16S rRNA database ((Release 138).

Alpha-diversity indices were calculated using mothur [21]. The richness and diversity of each sample were evaluated with the Sobs index and Shannon index, respectively. Wilcoxon rank-sum test was used to compare the alpha-diversity index between the pre-treatment group and the post-treatment group, and the Sob index and Shannon index were used to construct the rarefaction curves for comparison between the two groups. Hierarchical clustering analysis was performed on all samples based on Bray–Curtis distances and unweighted pair-group method with arithmetic means (UPGMA) algorithm. Species differences between the two groups were analyzed at the genus level using the Wilcoxon rank-sum test based on the microbial community abundance data. Linear discriminant analysis (LDA) effect size (LEfSe) algorithm was used to detect the microbial markers that contributed the most to the difference between the two groups. Q-value (*P*-value adjusted by false discovery rate (FDR)) and *P*-value < 0.05 were considered significant in this study.

## Results

### Clinical characteristics of the study population

A total of 37 patients were enrolled in this study. Thirty-seven pairs of vaginal microbial samples were obtained before and 3 months after FU treatment, with a total of 74 samples. After PCR amplification, the target bands of the PCR products were too weak or undetectable in 6 samples for subsequent experiments. Therefore, only 68 samples were finally sequenced, including 32 samples before treatment and 36 samples after treatment. The HR-HPV subtype with the highest infection rate in the study cohort was HPV16 (37.5%), followed by HPV52 (21.9%), and HPV51 (15.6%). At the 6-month follow-up, 24 patients were cleared of HR-HPV (defined as negative for all HR-HPV subtypes), with a clearance rate of 75.0% (24/32), while all 32 patients had cytology results of negative for intraepithelial lesion or malignancy (NILM). The clinical characteristics of patients are shown in Table 1.

**Table 1:** Clinical characteristics of patients (n = 32)

Characteristic	Value
Age(years)	40.6 ± 6.7 (26–53)
Gravidity	3.6 ± 1.5 (1–6)
Parity	1.8 ± 0.7 (0–3)
HPV infection types	
Single type infection with HPV16/18	11 (34.4%)
Co-infection with HPV16/18	3 (9.4%)
Single type infection without HPV16/18	13 (40.6%)
Co-infection without HPV16/18	5 (15.6%)
Cytology results (pre-treatment)	
NILM	25 (78.1%)
ASCUS	5 (15.6%)
LSIL	2 (6.3%)
HR-HPV outcome (at the 6-month follow-up)	
Cleared	24 (75.0%)
Non-cleared	8 (25.0%)

*NILM* negative for intraepithelial lesion or malignancy, *ASCUS* atypical squamous cells of undetermined significance, *LSIL* low-grade squamous intraepithelial lesion, *HR-HPV* high-risk human papillomavirus

### Sequencing results

A total of 11,957,834 optimized reads were obtained from 68 samples, with an average read lengths of 425 bp. For normalization, the reads in each sample were randomly subsampled to the lowest number of 108,824 in sample A19 (post-treatment group), and a total of 9784 ASVs were obtained.

### Vaginal microbiota richness and diversity

**Fig. 1:**
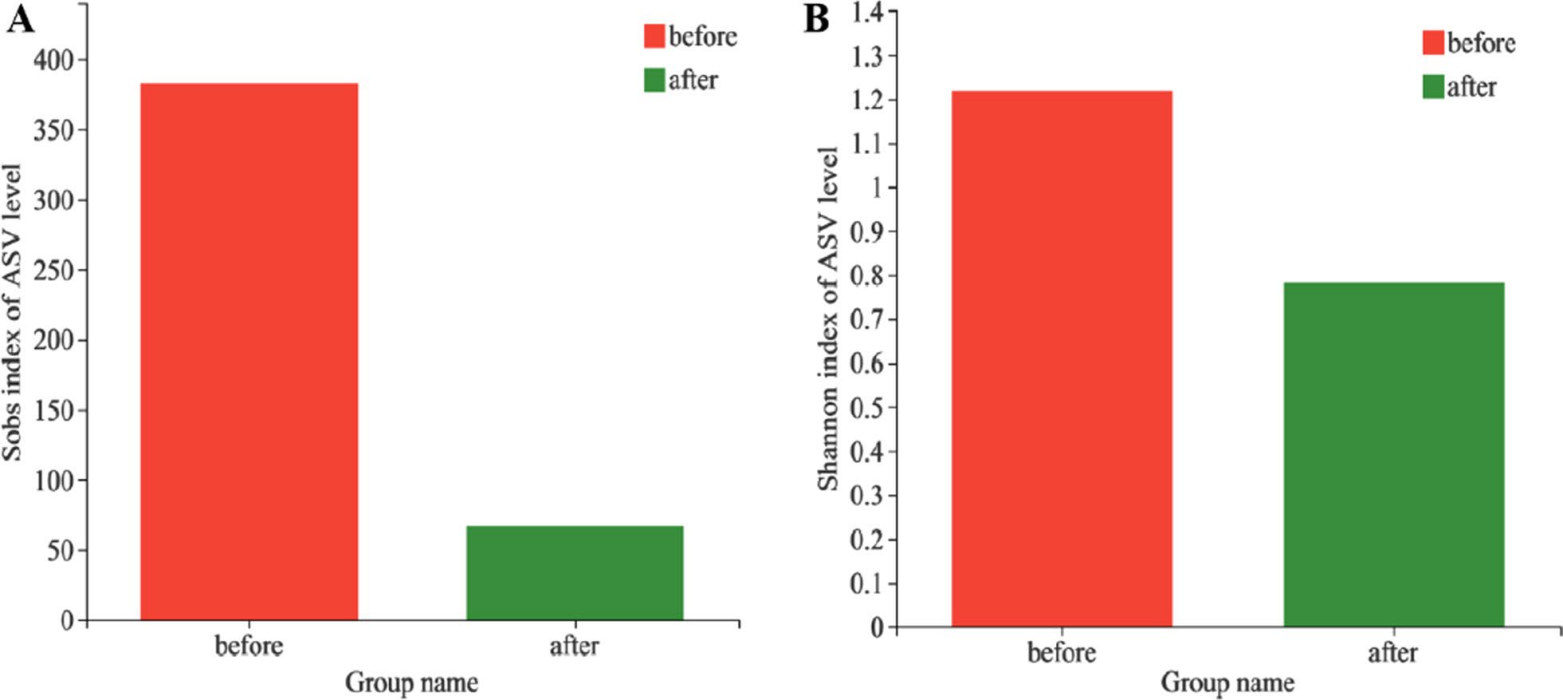
Vaginal microbial richness and diversity in two groups. **A** Sobs index. **B** Shannon index. Wilcoxon rank-sum test was used to compare differences between the two groups

At the ASV level, microbial richness and diversity were estimated using Sobs index and Shannon index, respectively. There was no significant difference in the Sobs index between the pre-treatment group and the post-treatment group (382.7 ± 1172.7 vs. 67.0 ± 50.6, *P* = 0.138), and no significant difference was found in the Shannon index between the two groups (1.2 ± 1.8 vs. 0.8 ± 0.7, *P* = 0.199) (Fig. 1).

**Fig. 2:**
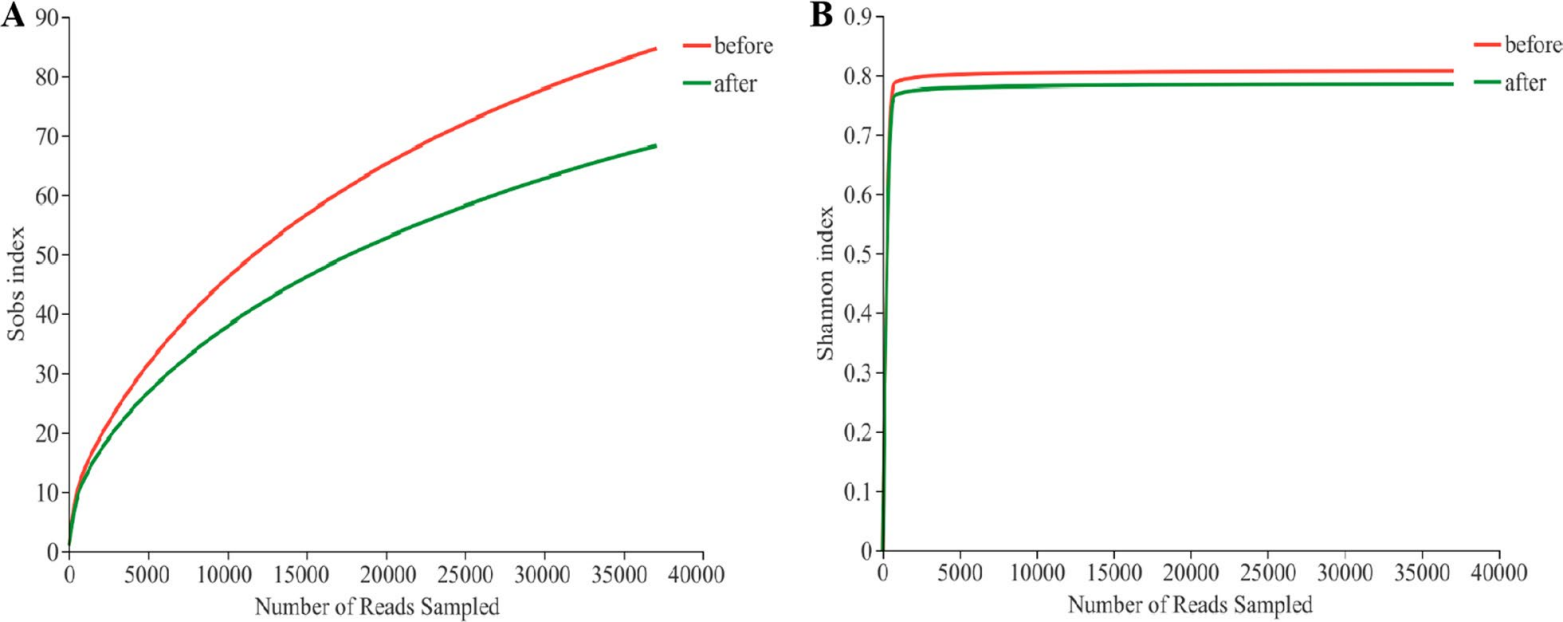
Rarefaction curves based on Sobs index and Shannon index in two groups. **A** Rarefaction curve based on Sobs index. **B** Rarefaction curve based on Shannon index

Rarefaction curves were constructed based on the Sobs index and Shannon index, respectively. The rarefaction curve based on the Shannon index reached a flat state, indicating that most of the microbial diversity had been generated, while the microbial diversity before treatment was higher than that after treatment. The rarefaction curve based on the Sobs index also indicated that the microbial richness before treatment was higher than that after treatment (Fig. 2).

### Taxonomy of the vaginal microbiota in two groups

**Fig. 3:**
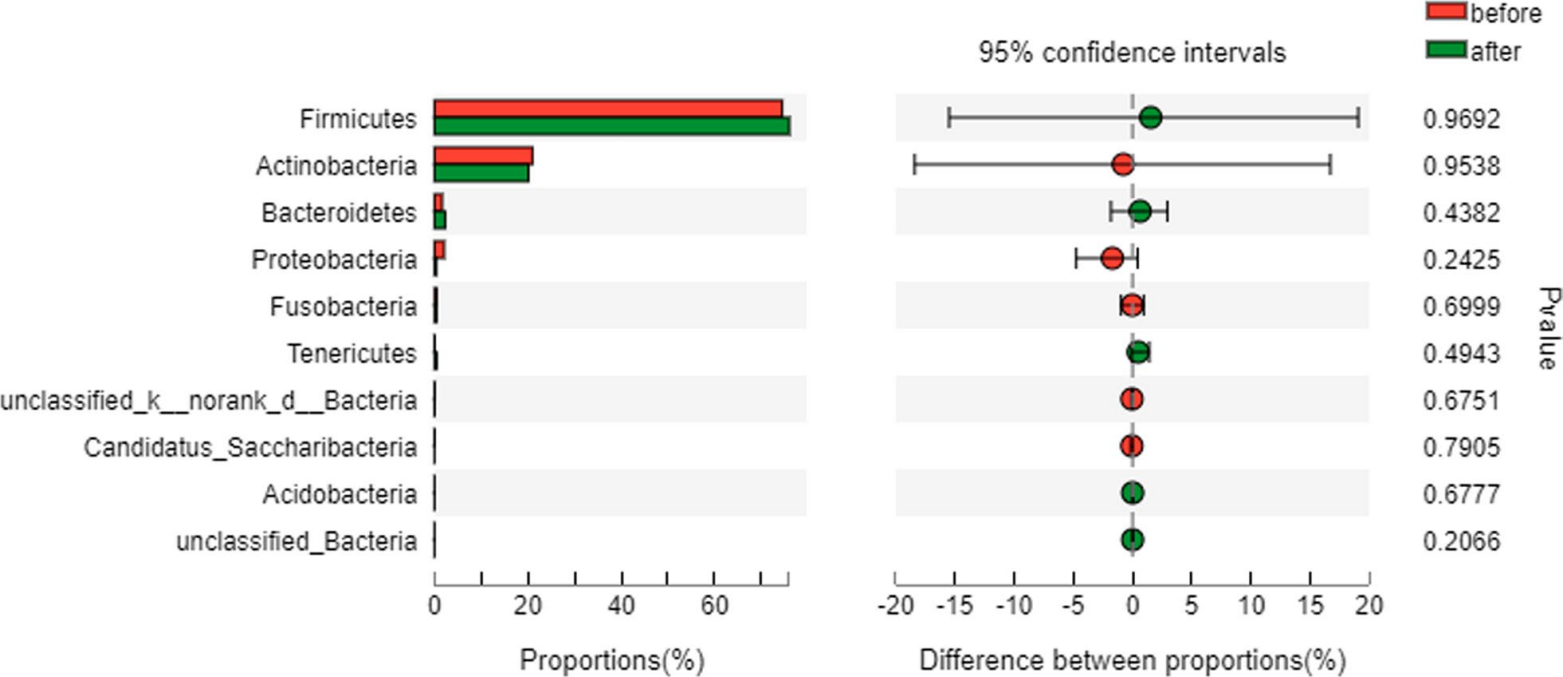
Comparison of the top 10 bacterial abundances between the two groups (phylum level)

Overall, 35 bacterial phyla were recovered across all samples, and *Firmicutes* was the most abundant phylum, followed by *Actinobacteria*, *Proteobacteria*, *Bacteroidetes*, and *Fusobacteria*. *Firmicutes* accounted for 74.47% and 76.00% of the pre-treatment group and the post-treatment group, respectively, and there was no significant difference between the two groups (*P* = 0.969). Moreover, there was no significant difference between the two groups in the abundance of the top 10 bacteria at the phylum level (*P* > 0.05) (Fig. 3).

**Fig. 4:**
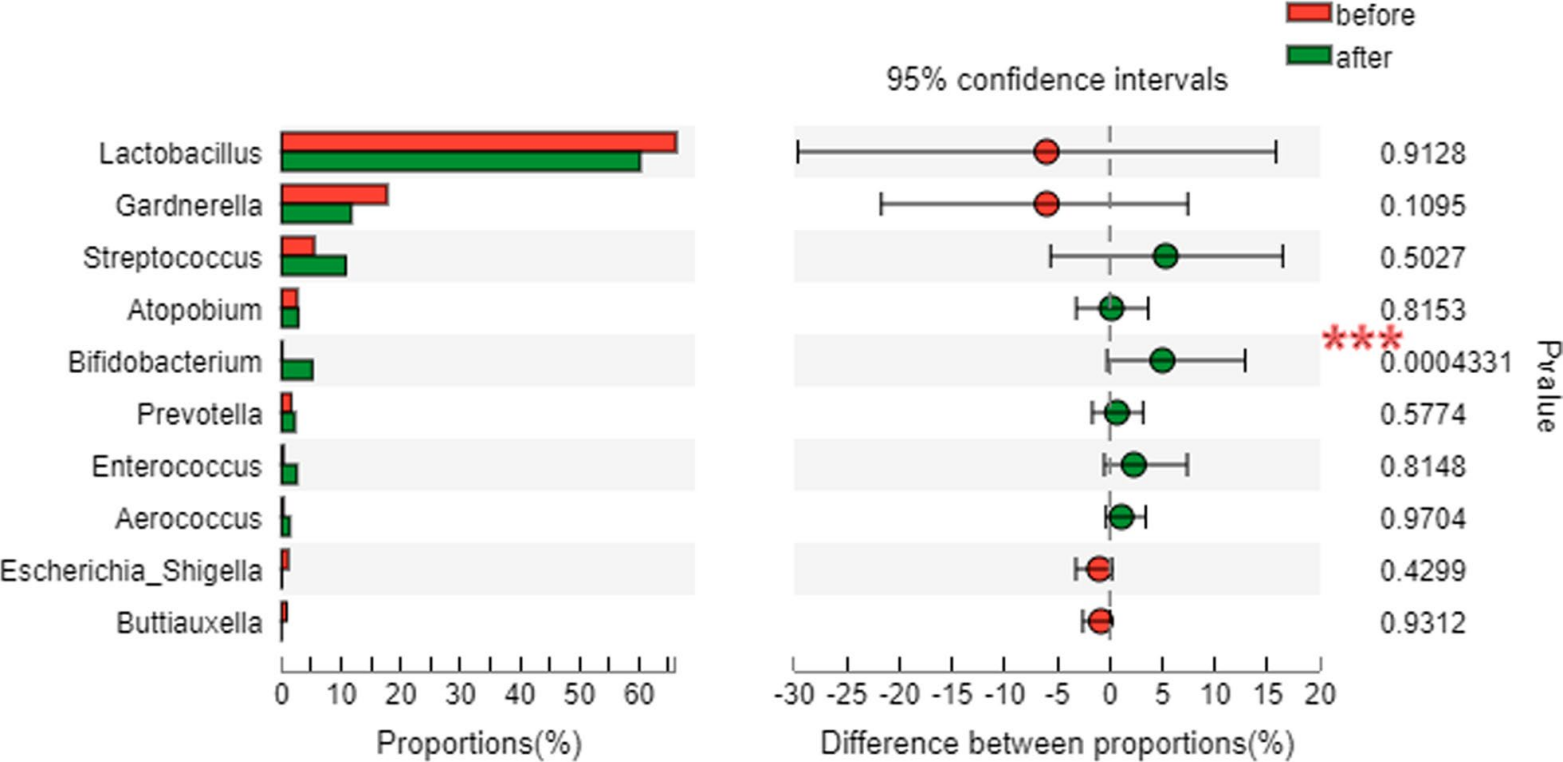
Comparison of the top 10 bacterial abundances between the two groups (genus level). ^***^P < 0.001

At the genus level, a total of 802 taxa were found across all samples, with *Lactobacillus* being the most dominant genus overall, followed by *Gardnerella*, *Streptococcus*, *Bifidobacterium*, and *Atopobium*. *Lactobacillus* accounted for 66.22% and 60.20% of the pre-treatment group and the post-treatment group, respectively, and there was no significant difference between the two groups (*P* = 0.913). Among the top 10 bacteria in abundance, *Bifidobacterium* accounted for 0.14% and 5.14% of the pre-treatment group and the post-treatment group, respectively. The abundance of *Bifidobacterium* after treatment was significantly higher than that before treatment (*P* < 0.001) (Fig. 4).

### Identification of vaginal microbial markers correlated with FU

**Fig. 5:**
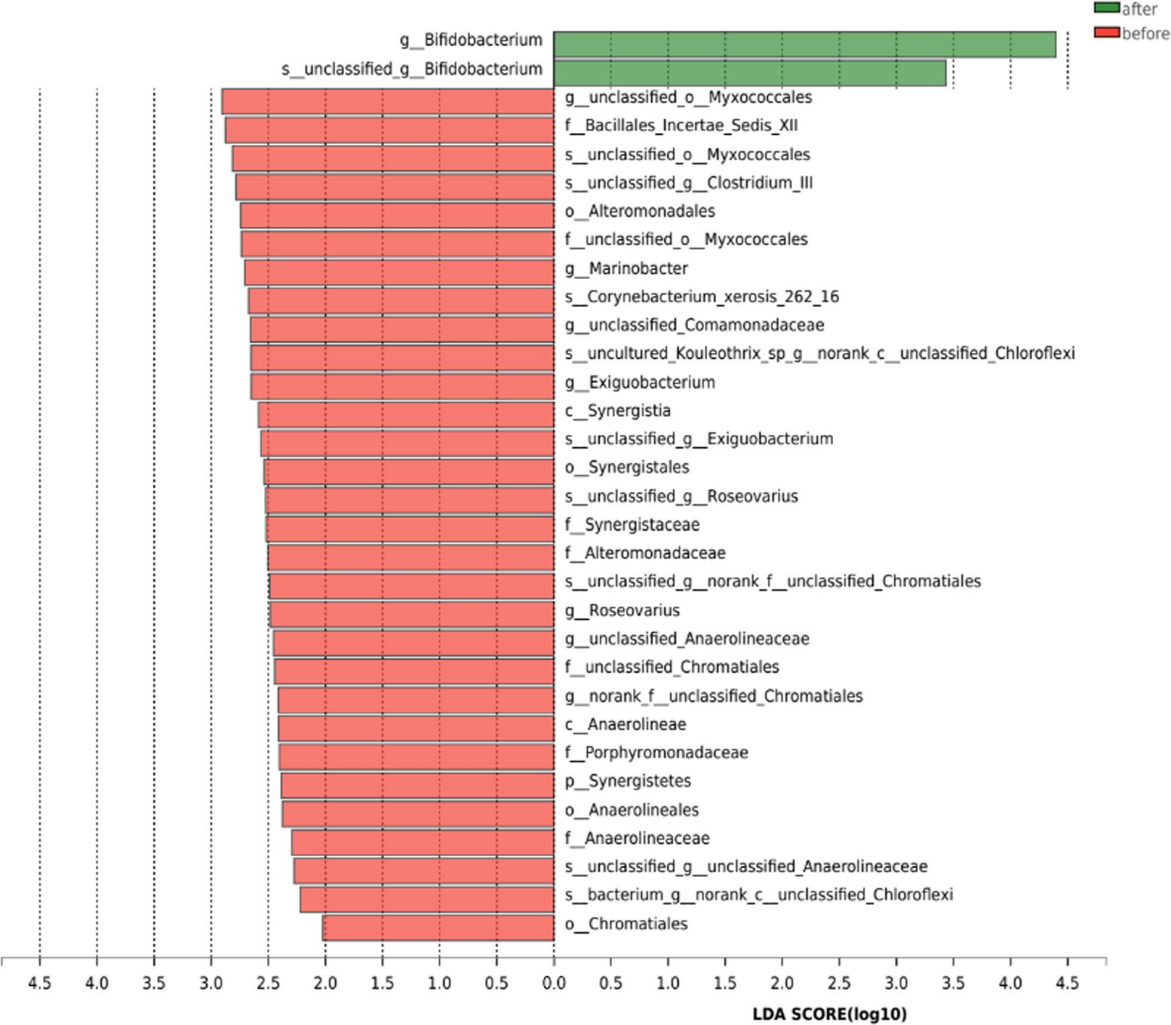
The unique taxa and microbial markers for the two groups. Shown is a histogram of LDA scores computed for features differentially abundant in the two groups

We used the LEfSe model to identify microbial markers related to FU treatment (Fig. 5). The threshold for the logarithmic LDA model score for discriminative features in this study was 2.0 (*P* < 0.01). FU treatment was strongly associated with *Bifidobacterium* at the genus level (LDA = 4.40, *P* = 0.000).

### Characteristics of vaginal microbial community types

**Fig. 6:**
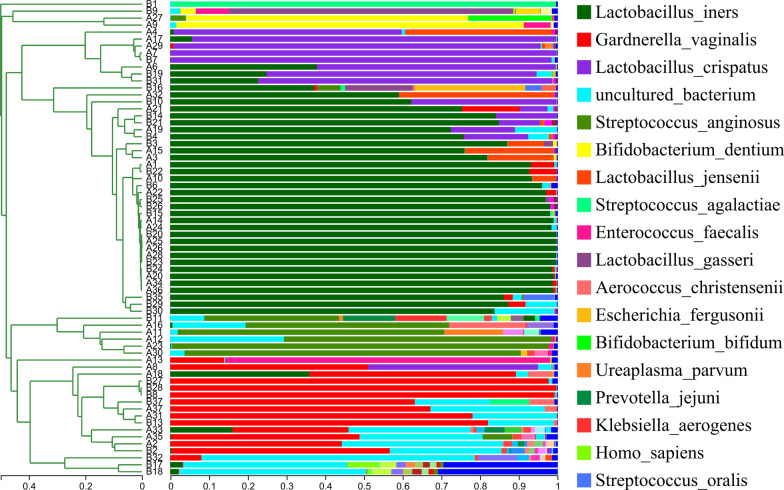
Hierarchical clustering tree of all samples at the species level. Based on Bray–Curtis distances and unweighted pair-group method with arithmetic means (UPGMA) algorithm

**Fig. 7:**
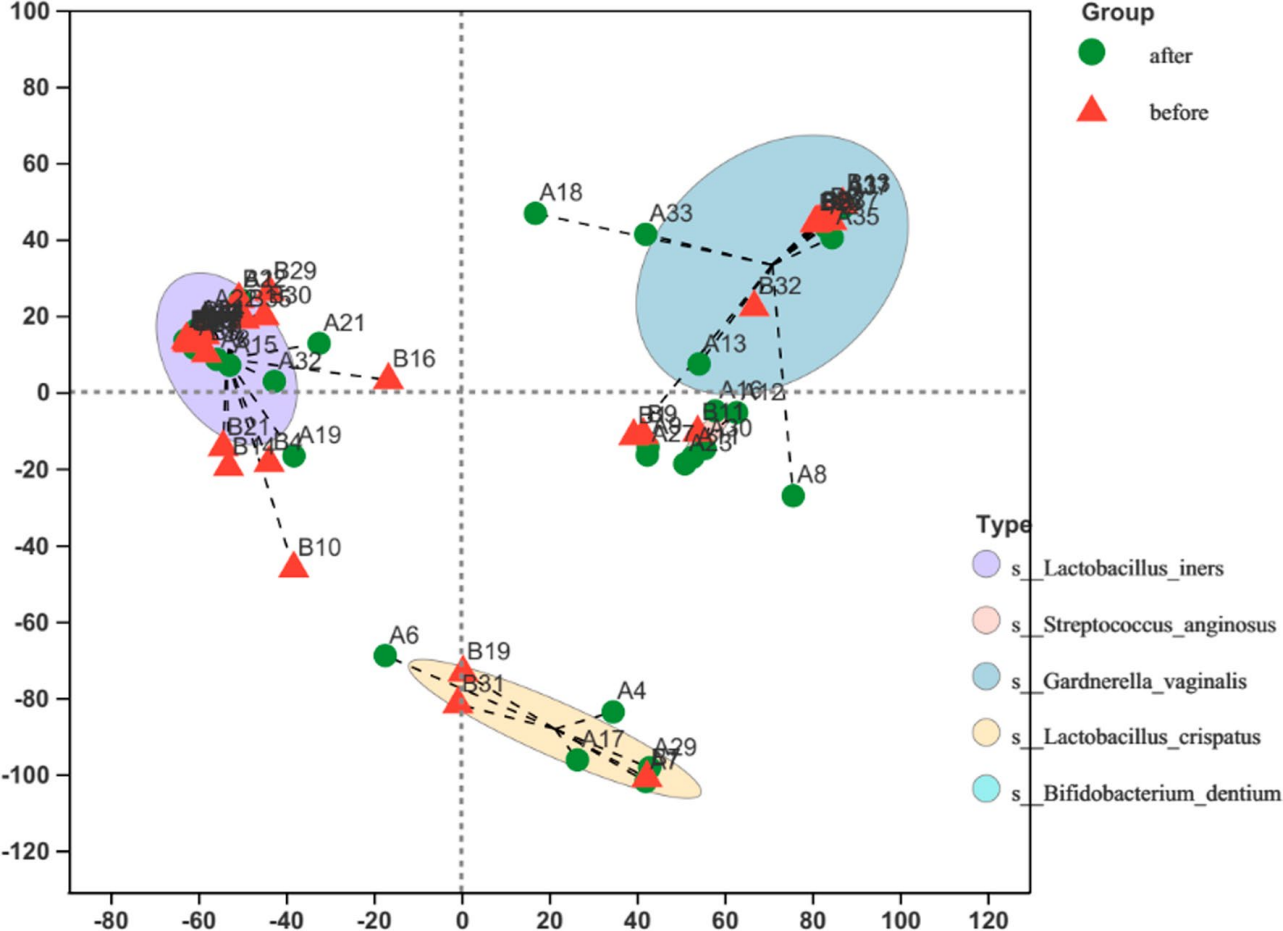
Typing analysis of all samples at the species level. Based on Jensen-Shannon distance

**Fig. 8:**
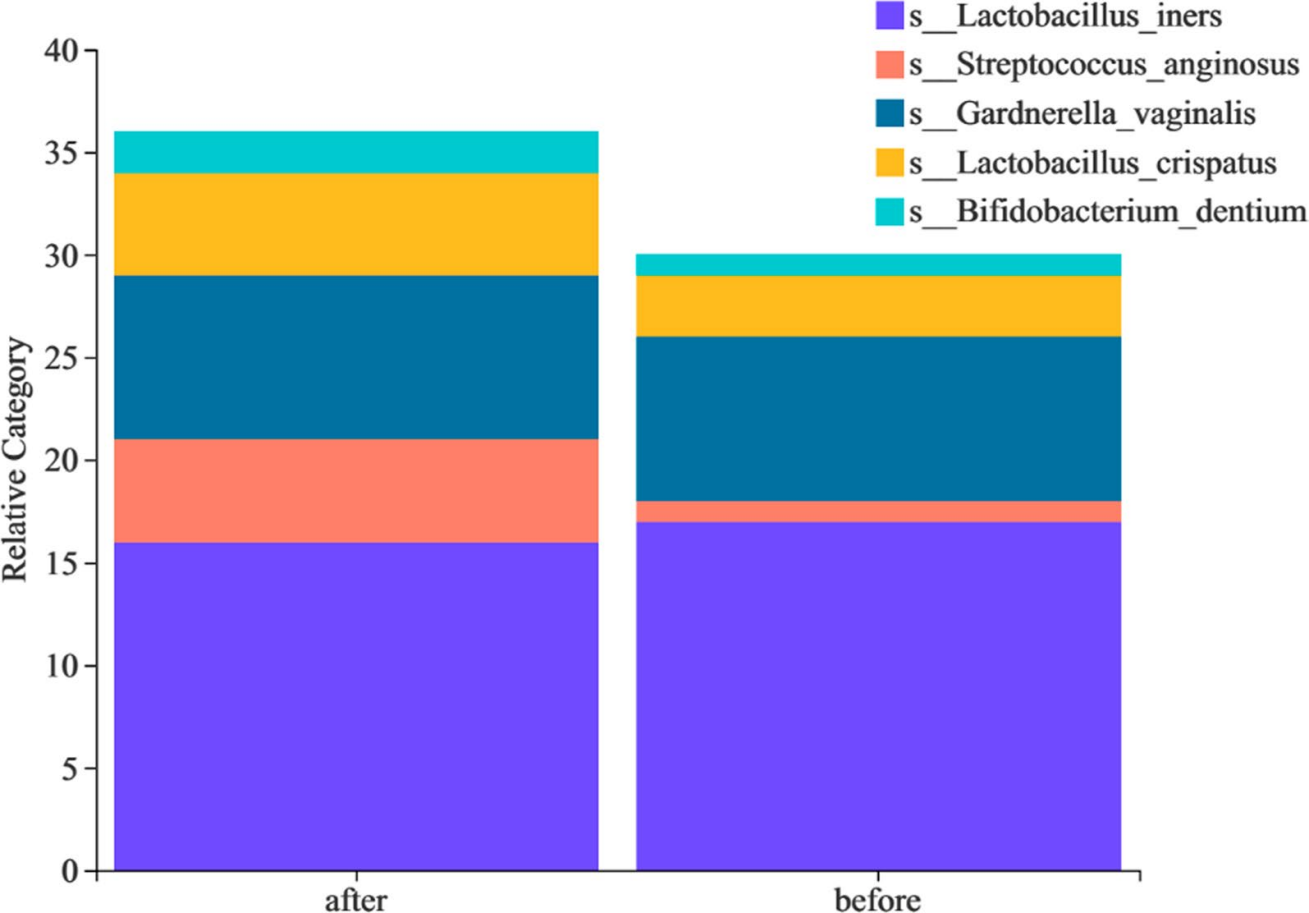
Typing histogram of the two groups at the species level

At the species level, hierarchical clustering analysis revealed that all samples clustered into five major community types: dominated by *Lactobacillus iners*, dominated by *Streptococcus anginosus*, dominated by *Gardnerella vaginalis*, dominated by *Lactobacillus crispatus*, and dominated by *Bifidobacterium dentiu* (Fig. 6). However, the pre-treatment and post-treatment samples cannot be clearly separated (Fig. 7). The community type dominated by *Lactobacillus iners* accounted for the highest proportion in the pre-treatment group and post-treatment group (Fig. 8).

## Discussion

In this study, we applied the 16S rRNA gene sequencing approach to a longitudinal cohort of patients before and after FU treatment to analyze the changes in the vaginal microbiome. A significant increase in *Bifidobacterium* abundance was observed in patients with HR-HPV infection-related LSIL at 3-month follow-up after FU treatment. Meanwhile, we found that the richness and diversity of vaginal microbiota decreased after FU treatment by rarefaction curves. *Lactobacillus* was still the most abundant genus in the two groups. However, at the species level, *Lactobacillus iners* was the predominant bacterium in the two groups.

The alpha-diversity index was widely used in microbial research. Several studies have suggested that HPV infection and progression of cervical lesions were associated with increased vaginal microbial diversity [22, 23]. Chen et al. [22] compared the Chao index and Shannon index in the HPV-negative group, HPV-positive group, LSIL group, high-grade squamous intraepithelial lesion (HSIL) group, and CC group. The results showed that the means of Chao and Shannon indices in the HPV-negative group was significantly lower than those in the HPV-positive group, while the values of Chao index and Shannon index in the CC group were the highest. However, other studies did not find a relationship between the alpha-diversity index and HPV infection and cervical lesions. Chalifour et al. [3] found no significant difference in the Shannon index between the HPV-negative group, HPV-positive group, and different stages of cervical lesions. In this study, we compared the Sobs index and Shannon index of the pre-treatment group and post-treatment group, and the results showed that the difference was not statistically significant (*P* > 0.05). However, from the rarefaction curves based on Sobs index and Shannon index, it could be found that the microbial richness and diversity before FU treatment were higher than those after FU treatment. Moreover, the HR-HPV clearance rate in the study cohort was 75.0% (24/32) at the 6-month follow-up. Pinder et al. [24] compared the efficacy of thermal ablation with cryotherapy or loop excision in a randomized controlled trial, showing that at the 6-month follow-up, the HR-HPV-negative rates were 40%, 42% and 47% in the cryotherapy, thermal ablation and LEEP treatment groups, respectively. In terms of the efficacy of FU, our results showed that FU was not inferior to other modalities. The microbial sequencing results suggested that the decrease in vaginal microbial diversity may be associated with the clearance of HR-HPV infection, and FU therapy contributed to the decrease of vaginal microbial diversity in patients.

Ravel et al. [25] classified vaginal microbiota into 5 major types, termed community state types (CSTs), based on vaginal bacterial species composition and abundance in reproductive-aged women. *Lactobacillus crispatus*, *Lactobacillus gasseri*, *Lactobacillus iners*, and *Lactobacillus jensenii* were the dominant bacteria in CST I, II, III, and V, respectively, while CST IV had a lower proportion of *Lactobacillus* and a higher proportion of strict and facultative anaerobes. We used hierarchical clustering and microbiota typing analysis to cluster the microbial communities of all samples into 5 types, and found that the dominant microbial community before and after FU treatment was dominated by *Lactobacillus iners*. Our results were consisted with other studies. A meta-analysis showed that vaginal microbiota dominated by *Lactobacillus iners* were significantly positively associated with HPV infection, cervical dysplasia and CC compared with *Lactobacillus crispatus* [12]. Many studies have found that the vaginal microbiota dominated by *Lactobacillus crispatus* (CST I) was associated with vaginal health status, whereas the vaginal microbiota dominated by *Lactobacillus iners* (CST III) was more common in patients with HPV infection or cervical lesions [26–28]. *Lactobacillus iners* can persist under the drastically changing vaginal environment due to its ability to respond and regulate its genomic functions [29], which may be the reason why FU therapy failed to alter the state of the vaginal microbial community.

We also found that *Bifidobacterium* at the genus level contributed the most to the difference between the pre-treatment group and post-treatment group, and the abundance of *Bifidobacterium* after FU treatment was significantly higher than that before FU treatment. *Bifidobacterium* is among the dominant bacterial populations in the human gastrointestinal tract (GIT), and also colonizes the vagina and oral cavity [30]. Studies have confirmed that *Bifidobacterium* in the GIT plays an important role in human physiology and nutrition [31]. However, there were few studies on the characteristics of *Bifidobacterium* in the vagina. Schellenberg et al. isolated vaginal *Bifidobacterium* with a ‘‘double-strong’’ phenotype of acid- and hydrogen peroxide (H_2_O_2_)- production [32], and the acid-producing property was thought to be responsible for maintaining vaginal microbial homeostasis. Freitas et al. found that lactic acid was produced by all vaginal *Bifidobacterium* isolates, but only 32% of isolates produced hydrogen peroxide [33]. These findings suggested that *Bifidobacterium* may be the dominant member of certain vaginal microbial communities, and may have protective or health-promoting effects in the vagina analogous to *Lactobacillus* by producing lactic acid [34].

Chao et al. [35] investigated the potential vaginal microbiome biomarkers for HSIL, and found that a paucity of *Bifidobacterium*, *Dialister*, *unidentified Prevotellaceae*, *Faecalibacterium*, and *Bacteroides* was related with HSIL. The relative abundance of *Bifidobacterium* being under 0.01% maybe a good predictor of HSIL in HPV16 and/or 18 infected individuals. Cha et al. [36] analyzed the antiviral activity of *Bifidobacterium adolescentis* SPM1005-A in the SiHa cervical cancer cell line expressing HPV 16, and found that the *Bifidobacterium* strain had antiviral activity through suppression E6 and E7 oncogene expression. Abdolalipour et al. [37] found that the intravenous or oral administration of *Bifidobacterium bifidum* can effectively induce antitumor immune responses and inhibit tumor growth in C57BL/6 mice bearing transplanted TC-1 cell of HPV-related tumor, expressing HPV 16 E6/E7 oncogenes. Our findings suggested that the increased abundance of *Bifidobacterium* in the vaginal microbiota may be associated with the clearance of HR-HPV infection, and FU treatment may contribute to the increased abundance of *Bifidobacterium*. However, studies on the role of *Bifidobacterium* in vaginal microbiota, HPV infection and HPV-related cervical lesions were still very insufficient.

The strength of this study is that it preliminarily explored the changes in the vaginal microbiome after FU treatment in patients with HR-HPV infection-related LSIL. We found that the diversity of the vaginal microbiome was decreased and the abundance of *Bifidobacterium* was increased after FU treatment in a prospective cohort, which provided clues to the mechanism of FU in clearing HR-HPV infection. We also confirmed that the vaginal microbial community of patients with HR-HPV infection-related LSIL was dominated by *Lactobacillus iners.* The limitations of this study were the small sample size and lack of a control group. Further studies with larger sample size and longer follow-up time are needed to assess the correlation between vaginal microbiome dynamics and FU treatment, HR-HPV infection clearance and LSIL lesions regression.

## Conclusion

This work investigated the effect of FU treatment on the vaginal microbial composition. *Bifidobacterium* as a biomarker of vaginal microbial differences was associated with FU treatment, and increased *Bifidobacterium* abundance may be associated with HR-HPV infection clearance. This suggests that the characteristics and roles of *Bifidobacterium* in the vaginal microbial community deserve further study.

## Data Availability

All reads obtained were submitted to NCBI Sequence Read Archive (SRA) under the accession number PRJNA826816 (https://www.ncbi.nlm.nih.gov/sra/PRJNA826816).

## Abbreviations

FU: Focused ultrasound
HR-HPV: High-risk human papillomavirus
HPV: Human papillomavirus
LSIL: Low-grade squamous intraepithelial lesion
CC: Cervical cancer
STI: Sexually transmitted infections
LEfSe: Linear discriminant analysis (LDA) effect size
ASV: Amplicon sequence variant
HSIL: High-grade squamous intraepithelial lesion
CST: Community state type
GIT: Gastrointestinal tract
TCT: ThinPrep cytology test
